# Validity of a continuous metabolic syndrome score as an index for modeling metabolic syndrome in children and adolescents: the CASPIAN-V study

**DOI:** 10.1186/s13098-017-0291-4

**Published:** 2017-11-09

**Authors:** Ramin Heshmat, Motahar Heidari, Hanieh-Sadat Ejtahed, Mohammad Esmaeil Motlagh, Armita Mahdavi-Gorab, Hasan Ziaodini, Majzoubeh Taheri, Gita Shafiee, Shaghayegh Beshtar, Mostafa Qorbani, Roya Kelishadi

**Affiliations:** 1 Chronic Diseases Research Center, Endocrinology and Metabolism Population Sciences Institute, Tehran University of Medical Sciences, Tehran, Iran; 2 Child Department of Pediatrics, Child Growth and Development Research Center, Research Institute for Primordial Prevention of Non-communicable Disease, Isfahan University of Medical Sciences, Isfahan, Iran; 3 Obesity and Eating Habits Research Center, Endocrinology and Metabolism Molecular -Cellular Sciences Institute, Tehran University of Medical Sciences, Tehran, Iran; 4 Department of Pediatrics, Ahvaz Jundishapur University of Medical Sciences, Ahvaz, Iran; 5 Department of Basic and Clinical Research, Tehran Heart Center, Tehran University of Medical Sciences, Tehran, Iran; 6 Health Psychology Research Center, Education Ministry, Tehran, Iran; 7 Office of Adolescents and School Health, Ministry of Health and Medical Education, Tehran, Iran; 8 Student Research Committee, School of Paramedical Sciences, Iran University of Medical Sciences, Tehran, Iran; 9 Non-communicable Diseases Research Center, Alborz University of Medical Sciences, Karaj, Iran; 10 Endocrinology and Metabolism Research Center, Endocrinology and Metabolism Clinical Sciences Institute, Tehran University of Medical Sciences, Tehran, Iran

**Keywords:** Metabolic syndrome, Continuous metabolic syndrome score, Validity, Pediatrics

## Abstract

**Background:**

The purpose of the present study was to assess the validity of continuous metabolic syndrome score (cMetS) for predicting metabolic syndrome (MetS) and to determine the cutoff values in a representative sample of Iranian children and adolescents.

**Methods:**

This national study was conducted among 3843 students, aged 7–18 years country during the fifth survey of a national school-based surveillance program. The cMetS was computed by standardizing the residuals of waist circumference, mean arterial blood pressure, high-density lipoprotein cholesterol, triglycerides, and glucose by regressing them according to age and sex and aggregating them. The optimal cut-off points of cMetS for predicting MetS were determined by the receiver operation characteristic (ROC) curve analysis in different gender and age categories.

**Results:**

Totally, 3843 students (52.3% boys) with average age of 12.45 years were assessed. The mean of cMetS increased according to elevating the number of MetS components. The overall cMetS cut-off point was 1.76 (sensitivity 93% and specificity 82%) in total pediatrics. The area under the ROC curve was 94%. The values for boys and girls were 1.79 and 2.72, respectively.

**Conclusions:**

cMetS performed highly accurate in predicting pediatrics with MetS in all gender and age groups and it appears to be a valid index in children and adolescents.

## Background

Metabolic syndrome (MetS) is characterized by co-existence of abdominal adiposity, elevated levels of blood pressure (BP), serum triglycerides (TG) and glucose, as well as low serum high density lipoprotein-cholesterol (HDL-C). MetS increases the morbidity and mortality of most chronic diseases [1, 2].

MetS is well defined in adults, however in the pediatric age group, there is no universal and uniform definition for MetS. Many studies in children and adolescents use the adult definitions with modified cutoff points for each component [3, 4].

The prevalence of MetS in children and adolescents has large variations in different studies. Therefore, modeling the relationship between risk factors and categorical variables of MetS and the use of discriminant function or multiple logistic regression analysis revealed controversial findings. Thus, a continuous value of metabolic risk score (cMetS) has been suggested for overcoming these limitations [5, 6].

Epidemiological studies that investigated cardio-metabolic risk factors among children and adolescents used various scores and statistical approaches to calculate the cMetS. Different variables are used in cMetS as indicators of obesity, lipids, glucose or insulin, BP and other components including smoking or physical activity. Various statistical approaches including principal component analysis, standardized residuals of Z-scores, and centile rankings have been applied because of the differences in variables included in the definition [5, 7, 8].

Utility of the cMetS is increasing in pediatric epidemiological research. The purpose of the present study is to construct cMetS in a large nationally representative sample of Iranian children and adolescents and to evaluate the efficacy and validity of this score in predicting the risk of MetS components. In addition, we determined cutoff points for cMetS score that were stratified by age and gender for identifying MetS in the pediatrics.

## Methods

### Study design and population

The data of present study were collected as a part of the fifth phase of a national school-based surveillance survey entitled “Childhood and Adolescence Surveillance and Prevention of Adult Non-communicable Disease” (CASPIAN V) in 2015. Totally 14,400 individuals aged 7–18 years participated in the survey. Sampling was conducted by multistage, stratified cluster sampling method from urban and rural areas of 30 provinces in Iran. Students with Iranian nationality, without any history of chronic diseases or surgery were included in this survey. Moreover, pregnant girls and pediatrics taking medications were excluded. 3843 students were randomly selected for biochemical test and fasting blood sample was obtained from them. Protocol of this study have been explained in detail previously [9].

The study was approved by the Research and Ethics Council of Isfahan University of Medical Sciences (code: 194049). After explaining the objectives and protocols of the study, written informed consent and verbal consent were obtained from all the parents and students, respectively.

### Anthropometric and laboratory measurements

Anthropometric measurements were performed by trained experts using calibrated instruments. Weight was measured to the nearest 0.1 kg while the subjects were minimally clothed. Height was measured in a standing position to the nearest 0.5 cm. Measurements were done without shoes [10]. Body mass index (BMI) was calculated as weight (kg) divided by square of height (m^2^). Waist circumference (WC) was measured us-ing non-elastic tape between the uppermost lateral border of right ilium and that of left ilium to the nearest 0.1 cm. Two measurements of blood pressure (BP) were done in the sitting position after 15 min of rest on the right arm using a standardized mercury sphygmomanometer. The first and fifth Korotkoff sounds were recorded as systolic blood pressure (SBP) and diastolic blood pressure (DBP), respectively. The mean of the two recorded values was considered as the subject’s BP. Mean arterial pressure (MAP) was calculated by this formula: [(SBP − DBP)/3] + DBP.

Fasting blood samples were drawn from participants after 12 to 14 h of overnight fast. Fasting blood glucose (FBG), total cholesterol (TC), low density lipoprotein-cholesterol (LDL-C), high density lipoprotein-cholesterol (HDL-C), and triglycerides (TG) were measured enzymatically by Hitachi auto-analyzer (Tokyo, Japan).

### Definition

#### Metabolic syndrome

In this study, MetS was defined according to the modified Adult Treatment Panel III (ATP III) criteria for the pediatric age group. MetS was defined as having at least three of the following: TG concentration of 150 mg/dL or greater; HDL-C concentration of 40 mg/dL or less; FBG concentration of 100 mg/dL or greater; abdominal obesity: waist to height ratio > 0.5; and either SBP or DBP greater than the 90th percentile for age, sex, and height [11].

Over weight and obesity in children were considered as a BMI between 85th percentile and 95th percentile and BMI greater than 95th percentile for age and sex according to WHO criteria, respectively. High LDL was defined as LDL > 110 mg/dL and High TC was defined as TC > 200 mg/dL.

#### Continuous mets (cMetS) score

The methodology of the cMetS score calculation was previously published in details [12]. In brief, the cMetS score was computed by standardizing the residuals (z-scores) of WC, MAP, HDL-C, TG, and FBG by regressing them according to age and sex. Because HDL-C is inversely related to MetS risk, it was multiplied by − 1. The cMetS score was calculated by aggregating the z scores for the individual variables. A higher cMetS score indicates a less favorable metabolic profile.

### Statistical analysis

Analyses were conducted using STATA version 11.0 (STATA Statistical Software: Release 11. STATA Corp LP. Package, College Station, TX, USA). All variables were checked for normality and presented as the mean ± standard deviation or number (percentage). The independent sample *t* test was used to compare continuous variables and the Chi square test was used to compare proportions according to age and sex groups. The ANOVA was used to compare continuous variables between more than two groups. To estimate valid cut-off values of cMetS score for predicting metabolic syndrome, the receiver operation characteristic (ROC) curve analysis was performed with an estimation of the sensitivity and specificity. Data were also analyzed separately for sex and age categories. The estimated cut-off values were determined using the minimum value of which represents the maximum sum of sensitivity and specificity. The area under curve (AUC) shows the ability of cMetS score cut-off points to discriminate students with and without metabolic syndrome. *P* values less than 0.05 were considered as statistically significant.

## Results

3843 students (52.3% boys) were participated in this study. The general characteristics of participants including anthropometric and biochemical measurements according to gender and age categories are presented in Table 1. Boys had an average higher weight, height and waist than girls in 7–10 and 15–18 years age groups (*P* < 0.001). However, girls had an average higher weight, height and BMI than boys in 11–14 years age group (*P* < 0.01). There were significant differences in mean SBP, DBP, MAP, FBS, TC and LDL-C between girls and boys in total participants (*P* < 0.05).

**Table 1:** Mean of cardiometabolic risk factors according to gender and age groups: the CASPIAN-V study

	Total	Boys	Girls	P value
7–10 years				
Weight	27.69 (8.80)	28.36 (9.56)	27.06 (7.95)	< 0.001
Height	130.05 (10.15)	130.87 (10.50)	129.27 (9.75)	< 0.001
Waist	59.55 (9.05)	60.03 (9.54)	59.09 (8.53)	< 0.001
SBP	93.99 (12.72)	93.85 (12.69)	94.12 (12.74)	0.46
DBP	60.82 (10.35)	60.63 (10.31)	61 (10.39)	0.22
FBS	91.67 (14.00)	92.31 (17.26)	91.05 (9.83)	0.12
TG	87.12 (45.64)	86.78 (49.06)	87.45 (42.09)	0.80
TC	154.72 (29.21)	155.43 (29.37)	154.03 (29.06)	0.41
HDL-C	47.08 (10.60)	47.59 (10.80)	46.59 (10.38)	0.11
MAP	71.88 (10.28)	71.70 (10.19)	72.04 (10.36)	0.26
BMI	16.18 (4.03)	16.36 (4.65)	16 (3.32)	0.002
WHtR	0.45 (.06)	0.45 (.06)	0.45 (.05)	0.36
LDL-C	90.21 (24.12)	90.48 (23.46)	89.95 (24.75)	0.7
11–14 years				
Weight	42.05 (13.11)	41.43 (13.60)	42.68 (12.56)	< 0.001
Height	148.85 (11.79)	148.43 (12.25)	149.28 (11.28)	0.007
Waist	67.47 (11.18)	67.68 (11.25)	67.25 (11.11)	0.15
SBP	99.43 (12.33)	99.16 (12.61)	99.71 (12.03)	0.1
DBP	63.94 (9.97)	63.93 (10.20)	63.95 (9.74)	0.92
FBS	91.77 (11.33)	92.18 (10.45)	91.33 (12.16)	0.12
TG	87.76 (44.37)	85.67 (41.60)	89.96 (47.01)	0.04
TC	154.07 (26.30)	153.29 (27.21)	154.89 (25.30)	0.21
HDL-C	46.04 (9.75)	46.59 (10.06)	45.46 (9.38)	0.01
MAP	75.77 (9.81)	75.68 (10.01)	75.87 (9.60)	0.45
BMI	18.66 (4.41)	18.48 (4.76)	18.85 (4.01)	0.002
WHtR	0.45 (.06)	0.45 (.06)	0.45 (.06)	0.002
LDL-C	90.47 (21.69)	89.56 (22.72)	91.43 (20.53)	0.07
15–18 years				
Weight	57.68 (15.43)	59.75 (16.83)	55.31 (13.27)	< 0.001
Height	164.01 (12.27)	167.71 (13.54)	159.80 (8.93)	< 0.001
Waist	74.66 (11.70)	76.38 (12.76)	72.68 (10)	< 0.001
SBP	105.25 (11.89)	106.55 (12.09)	103.76 (11.47)	< 0.001
DBP	67.44 (10.01)	68.21 (10.38)	66.57 (9.48)	< 0.001
FBS	91.44 (11.02)	91.66 (11.17)	91.14 (10.82)	0.45
TG	89.50 (45.94)	89.59 (47.29)	89.38 (44.07)	0.93
TC	152.53 (27.10)	150.18 (27.78)	155.75 (25.82)	0.001
HDL-C	45.43 (9.54)	44.39 (9.45)	46.86 (9.49)	< 0.001
MAP	80.05 (9.66)	80.98 (10.05)	78.98 (9.08)	< 0.001
BMI	21.21 (4.42)	20.94 (4.44)	21.51 (4.37)	< 0.001
WHtR	0.45 (0.06)	0.45 (0.06)	0.45 (0.06)	0.77
LDL-C	89.20 (22.31)	87.87 (22.56)	91.02 (21.84)	0.02
Total				
Weight	41.39 (17.11)	42.36 (18.23)	40.41 (15.82)	< 0.001
Height	146.56 (17.50)	148.15 (18.77)	144.93 (15.93)	< 0.001
Waist	66.72 (12.17)	67.65 (12.87)	65.76 (11.33)	< 0.001
SBP	99.17 (13.09)	99.55 (13.43)	98.77 (12.72)	< 0.001
DBP	63.83 (10.43)	64.08 (10.70)	63.57 (10.14)	0.004
FBS	91.65 (12.11)	92.06 (12.91)	91.20 (11.14)	0.026
TG	88.04 (45.18)	87.15 (45.52)	89.02 (44.78)	0.200
TC	153.85 (27.42)	152.96 (28.06)	154.83 (26.67)	0.035
HDL-C	46.19 (9.97)	46.21 (10.17)	46.16 (9.75)	0.862
MAP	75.61 (10.42)	75.91 (10.71)	75.31 (10.12)	0.001
BMI	18.51 (4.71)	18.48 (4.96)	18.53 (4.43)	0.565
WHtR	0.45 (0.06)	0.45 (0.06)	0.45 (0.06)	0.008
LDL-C	90.05 (22.60)	89.31 (22.90)	90.86 (22.26)	0.034

Data are expressed as mean (SD)

*BMI* body mass index; *DBP* diastolic blood pressure; *FBS* fasting blood sugar; *HDL-C* high-density lipoprotein cholesterol; *LDL-C* low-density lipoprotein cholesterol; *MAP* mean arterial pressure; *SBP* systolic blood pressure; *TC* total cholesterol; *TG* triglycerides; *WHtR* waist to height ratio

Totally, 9.4% of pediatrics were overweight and 11.4% were obese. Prevalence of overweight was higher in girls than boys (10.2% vs. 8.7%) and prevalence of obesity was higher in boys than girls (12.5% vs. 10.3%) (*P* < 0.01). Table 2 presents the prevalence of cardiometabolic risk factors in children and adolescents according to age and gender categories. The prevalence of MetS in total participants was 5% with no significant difference between boys and girls. The mean of cMetS according to the number of MetS components was shown in Table 3. Pediatrics with higher number of MetS components had higher cMetS in all gender and age categories (*P* < 0.001).

**Table 2:** Prevalence of cardiometabolic risk factors in Iranian children and adolescents: the CASPIAN-V study

	Total	Boys	Girls	P value
7–10 years				
Abdominal obesity	974 (20.4)	484 (20.7)	490 (20.0)	0.581
Overweight	393 (8.2)	182 (7.8)	211 (8.6)	0.289
Obese	530 (11.1)	298 (12.7)	232 (9.5)	< 0.001
High FBG	47 (4.1)	31 (5.5)	16 (2.7)	0.019
High TG	299 (26.1)	139 (24.6)	160 (27.5)	0.265
High LDL-C	208 (18.1)	97 (17.2)	111 (19.1)	0.403
High TC	72 (6.3)	37 (6.5)	35 (6.0)	0.709
Low HDL-C	275 (24.0)	123 (21.8)	152 (26.1)	0.085
Elevated systolic BP	229 (4.8)	89 (3.9)	140 (5.7)	0.003
Elevated diastolic BP	427 (9.0)	206 (9.0)	221 (9.1)	0.949
Elevated BP	515 (10.9)	239 (10.5)	276 (11.3)	0.340
Mets	55 (4.9)	27 (4.9)	28 (5.0)	0.966
Number of mets				
0	487 (43.6)	238 (43.2)	249 (44.1)	0.802
1	391 (35.0)	201 (36.5)	190 (33.6)	
2	183 (16.4)	85 (15.4)	98 (17.3)	
> 3	55 (5.0)	27 (4.9)	28 (5.0)	
11–14				
Abdominal obesity	1156 (20.9)	606 (21.7)	550 (20.1)	0.151
Overweight	567 (10.2)	260 (9.3)	307 (11.2)	0.019
Obese	675 (12.2)	372 (13.3)	303 (11.0)	0.011
High FBG	71 (4.3)	37 (4.4)	34 (4.2)	0.864
High TG	457 (27.6)	219 (25.9)	238 (29.4)	0.108
High LDL-C	282 (17.0)	147 (17.4)	135 (16.7)	0.710
High TC	74 (4.5)	40 (4.7)	34 (4.2)	0.605
Low HDL-C	428 (25.9)	206 (24.3)	222 (27.4)	0.151
Elevated systolic BP	158 (2.9)	86 (3.1)	72 (2.6)	0.323
Elevated diastolic BP	729 (13.2)	365 (13.1)	364 (13.4)	0.770
Elevated BP	775 (14.1)	388 (13.9)	387 (14.2)	0.760
Mets	85 (5.3)	46 (5.6)	39 (5.0)	0.598
Number of mets				
0	628 (39.0)	334 (40.4)	294 (37.5)	0.487
1	580 (36.0)	296 (35.8)	284 (36.3)	
2	316 (19.6)	150 (18.2)	166 (21.2)	
> 3	85 (5.3)	46 (5.6)	39 (5)	
15–18				
Abdominal obesity	842 (22.2)	460 (22.7)	382 (21.6)	0.421
Overweight	370 (9.7)	179 (8.8)	191 (10.7)	0.046
Obese	410 (10.8)	226 (11.1)	184 (10.3)	0.433
High FBG	43 (4.1)	28 (4.7)	15 (3.4)	0.319
High TG	309 (29.7)	183 (30.4)	126 (28.6)	0.538
High LDL-C	184 (17.7)	97 (16.1)	87 (19.8)	0.126
High TC	43 (4.1)	23 (3.8)	20 (4.5)	0.561
Low HDL-C	431 (41.4)	329 (54.7)	102 (23.2)	< 0.001
Elevated systolic BP	51 (1.4)	35 (1.7)	16 (0.9)	0.029
Elevated diastolic BP	294 (7.8)	175 (8.7)	119 (6.8)	0.031
Elevated BP	314 (8.3)	188 (9.3)	126 (7.2)	0.018
Mets	48 (4.8)	35 (6.0)	13 (3.1)	0.035
Number of mets				
0	328 (32.6)	134 (22.8)	194 (46.2)	< 0.001
1	386 (38.3)	250 (42.6)	136 (32.4)	
2	245 (24.3)	168 (28.6)	77 (18.3)	
> 3	48 (4.8)	35 (6)	13 (3.1)	
Total				
Abdominal obesity	2972 (21.1)	1550 (21.6)	1422 (20.5)	0.087
Overweight	1330 (9.4)	621 (8.7)	709 (10.2)	0.002
Obese	1615 (11.4)	896 (12.5)	719 (10.3)	< 0.001
High FBG	161 (4.2)	96 (4.8)	65 (3.8)	0.060
High TG	1065 (27.7)	541 (26.9)	524 (28.6)	0.228
High LDL-C	674 (17.5)	341 (16.9)	333 (18.2)	0.310
High TC	189 (4.9)	100 (5.0)	89 (4.9)	0.878
Low HDL-C	1134 (29.5)	658 (32.7)	476 (26.0)	< 0.001
Elevated systolic BP	438 (3.1)	210 (3.0)	228 (3.3)	0.255
Elevated diastolic BP	1450 (10.4)	746 (10.5)	704 (10.2)	0.510
Elevated BP	1604 (11.5)	815 (11.5)	789 (11.4)	0.877
Mets	188 (5)	108 (5.5)	80 (4.5)	0.174
Number of mets components				
0	1443 (38.7)	706 (35.9)	737 (41.7)	0.005
1	1357 (36.4)	747 (38.0)	610 (34.5)	
2	744 (19.9)	403 (20.5)	341 (19.3)	
> 3	161 (5.1)	108 (5.5)	80 (4.5)	

Data are expressed as number (%)

Overweight: BMI; 85th–95th; obesity, BMI > 95th; low HDL: < 40 mg/dL (except in boys 15–19 y old, that cut-off was < 45 mg/dL); high LDL: > 110 mg/dL; high TG: 100 mg/dL; high TC: > 200 mg/dL; elevated FBS > 100 mg/dL; high blood pressure: > 90th (adjusted by age, sex, height)

*BP* blood pressure; *TG* triglycerides; *FBG* fasting blood glucose; *HDL-C* high-density lipoprotein-cholesterol; *TC* total cholesterol; *LDL-C* low-density lipoprotein cholesterol

**Table 3:** Mean of Continuous Metabolic Syndrome score by Mets and numbers of MetS components

		Total			Boys			Girls		
		cMetS score			cMetS score			cMetS score		
MetS		7–10	11–14	15–18	7–10	11–14	15–18	7–10	11–14	15–18
	Yes	4.4 ± 2.02	4.2 ± 1.62	4.2 ± 2.35	4.2 ± 2.41	4.2 ± 1.69	4.3 ± 2.45	4.6 ± 1.58	4.3 ± 1.56	3.8 ± 2.15
	No	− 0.4 ± 2.36	− 0.2 ± 2.3	− 0.3 ± 2.45	− 0.4 ± 2.58	− 0.4 ± 2.24	− 0.2 ± 2.52	− 0.3 ± 2.12	0.004 ± 2.34	− 0.5 ± 2.35
	P value	< 0.001	< 0.001	< 0.001	< 0.001	< 0.001	< 0.001	< 0.001	< 0.001	< 0.001
MetS components	0	− 1.7 ± 1.74	− 1.7 ± 1.78	− 2.2 ± 1.83	− 1.9 ± 1.84	− 1.8 ± 1.8	− 2.6 ± 1.8	− 1.6 ± 1.62	− 1.5 ± 1.75	− 1.9 ± 1.81
	1	0.1 ± 1.72	0.1 ± 1.82	− 0.2 ± 1.78	0.08 ± 1.79	− 0.09 ± 1.69	− 0.3 ± 1.82	0.1 ± 1.65	0.3 ± 1.92	− 0.1 ± 1.71
	2	2.1 ± 2.44	2.2 ± 1.63	1.8 ± 2.13	2.4 ± 3.06	2.09 ± 1.57	1.7 ± 2.21	1.9 ± 1.7	2.3 ± 1.68	2.04 ± 1.95
	+3	4.4 ± 2.02	4.2 ± 1.62	4.2 ± 2.35	4.2 ± 2.41	4.2 ± 1.69	4.3 ± 2.45	4.6 ± 1.58	4.3 ± 1.56	3.8 ± 2.15
	Total	− 0.1 ± 2.57	0.03 ± 2.48	− 0.1 ± 2.64	− 0.2 ± 2.76	− 0.1 ± 2.45	0.03 ± 2.74	− 0.1 ± 2.37	0.2 ± 2.5	− 0.4 ± 2.46
	P value	< 0.001	< 0.001	< 0.001	< 0.001	< 0.001	< 0.001	< 0.001	< 0.001	< 0.001

Data are expressed as mean ± SD

Metabolic syndrome: ATP-III criteria; Abdominal obesity: WC > 90th percentile; Low HDL: HDL < 40 mg/dL (except in boys 15–19 years old, that cut-off was < 45 mg/dL); High TG: TG > 100 mg/dL; High FBG: FBG > 100 mg/dL; High blood pressure: BP > 90th (adjusted by age, sex, height)

**Fig. 1:**
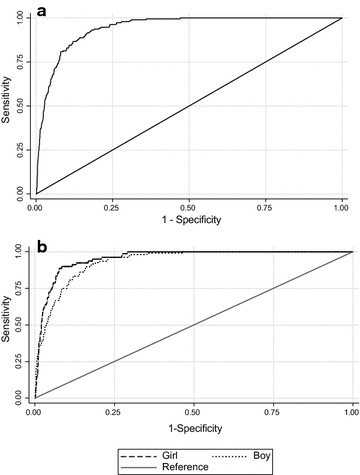
ROC curves for metabolic syndrome. **a** ROC curves for metabolic syndrome in total population. **b** ROC curves for metabolic syndrome by sex. *ROC* receiver operating characteristic

Table 4 shows the cut-off points of cMetS to predict MetS with sensitivity, specificity and AUC for gender and age categories. In boys, cMetS values for predicting MetS in 7–10, 11–14 and 15–18 years age groups were 1.95, 1.70 and 2.44, respectively. These scores for girls were 2.80, 2.72 and 2.34 in different age categories, respectively. The overall cMetS cut-off point was 1.76 (sensitivity 93%, specificity 82% and AUC 94%) in total pediatrics, 1.79 (sensitivity 92%, specificity 82% and AUC 93%) in boys and 2.72 (sensitivity 90%, specificity 91% and AUC 95%) in girls. In all groups, cMetS performed highly accurate in predicting students with MetS (90 < AUC < 100%). The ROC curves of the cMetS for MetS stratified by gender are displayed in Fig. 1.

**Table 4:** Receiver operator curve for cMetS score for identifying children with MetS

	cMetS score cut-off points (95% CI)	Sensitivity (95% CI)	Specificity (95% CI)	AUC (95% CI)
7–10 years				
Boy	1.95 (1.23–2.68)	96 (92–100)	86 (80–92)	93 (91–96)
Girl	2.80 (2.04–3.56)	93 (86–99)	93 (87–99)	97 (95–99)
Total	1.97 (1.51–2.43)	96 (91–100)	86 (83–90)	95 (94–97)
11–14 years				
Boy	1.70 (1.09–2.30)	98 (90–100)	82 (75–89)	95 (93–97)
Girl	2.72 (2.07–3.36)	92 (85–99)	90 (82–98)	95 (93–97)
Total	1.75 (0.78–2.72)	96 (88–100)	81 (70–92)	95 (93–97)
15–18 years				
Boy	2.44 (0.92–3.97)	80 (66–94)	86 (71–99)	90 (86–94)
Girl	2.34 (0.90–3.96)	82 (67–95)	87 (71–99)	93 (88–98)
Total	2.38 (0.68–4.07)	81 (68–94)	87 (71–99)	92 (88–95)
Boy (7–18 years)	1.79 (1.16–2.41)	92 (86–97)	82 (76–89)	93 (91–95)
Girl (7–18 years)	2.72 (2.31–3.12)	90 (83–97)	91 (88–95)	95 (94–97)
Total (7–18 years)	1.76 (1.16–2.36)	93 (86–100)	82 (75–88)	94 (93–95)

Metabolic syndrome: ATP-III criteria; Abdominal obesity: WC > 90th percentile; Low HDL: HDL < 40 mg/dL (except in boys 15–19 years old, that cut-off was < 45 mg/dL); High TG: TG > 100 mg/dL; High FBG: FBG > 100 mg/dL; High blood pressure: BP > 90th (adjusted by age, sex, height)

*CI* confidence interval; *AUC* area under curve, shown as percentage

## Discussion

In the present study, we determined the age- and gender- specific optimal cutoff points of cMetS in correlation to MetS and its validity in a large population-based sample of children and adolescents.

Our results obviously demonstrated an association between cMetS and MetS components. In addition, by ROC analyses, we indicated the optimal cutoff points for cMetS in various age groups according to gender. Results of the ROC analysis demonstrated a cMetS of 1.76 as the optimal cutoff point in 7–18 years old subjects in both gender. The area under the curve (AUC) for this index was 94%, which shows cMetS score is highly accurate and sensitive enough in predicting the presence of MetS in children and adolescents. The optimal cMetS cut-off points for boys and girls were 1.79 and 2.72, respectively in the total study. Our study showed that the cMetS was higher in subjects with MetS and it enhanced with increasing number of MetS factors. Those with equal or more than three risk factors had the highest cMetS. Our findings are consistent with some previous findings and support the use of cMetS in epidemiological surveys in children and adolescents [13–16].

Kelly et al. [17] have demonstrated significant relationship between childhood MetS, defined by cMetS, and adult cardiovascular risk. Okosun et al. [18] have shown an association between the cMetS and having 1 to 5 components of MetS.

Because of the lack of adequate and accepted criteria for the definition of MetS in pediatric age group, and increasing prevalence rate of MetS in children and adolescents, metabolic risk scores have been used to indicate clustering of metabolic risk factors. cMetS is calculated from continuous variables for the MetS components. cMetS in childhood has been correlated with the progress of MetS in young adulthood, which confirms the public health relevance of the investigation on cMetS. For calculating cMetS, it is assumed that all components of MetS are equally important and responsible in determining the cardiovascular risk factors [14, 19, 20].

The results of our previous study confirmed the validity of the cMetS score in a population-based sample of Iranian children and adolescents. Our findings serve as confirmatory evidence that cMetS can be used as an appropriate index for investigating the association between potential risk factors and MetS in epidemiological studies in the pediatric population.

Compared with individual MetS components, cMetS can investigate the risk of metabolic abnormalities by a more practical approach. cMetS is more sensitive and less error- prone and might increase the statistical power compared with binary definition of MetS particularly at early stages of metabolic abnormalities [21, 22]. Therefore, the use of cMetS has been supported as an alternative to the categorical measures that are often used for MetS in epidemiological studies [5, 23]. However, the binary or categorical definition of MetS remains advantageous for clinical practice [24].

The cMetS score is sample-specific and this is one of the main limitations of this score. Thus, the mean cMetS obtained from this study cannot be generalized and compared to other studies unless the data distribution, the demographic characteristics, and the measures of central tendency and variability of data would be similar. cMetS cutoff points must be calculated and validated for each study population. In addition, comparison of results in various studies is difficult because of using of different variables and statistical approaches [25].

Eisenmann recommended five key metabolic syndrome variables in the calculation of the cMetS in the pediatric research. These variables include central obesity (waist circumference, body mass index or skin fold thickness), low HDL-C, elevated TG, elevated BP (systolic, diastolic or mean arterial pressure) and abnormal glucose metabolism (impaired fasting glucose, impaired glucose tolerance or HOMA). If the validity of cMetS would be confirmed as an index for modeling pediatric MetS, it can be used as simple and practical tool in future pediatric epidemiological research, clinical medicine, and public health surveys for prevention, diagnosis and management of MetS and its components in the pediatric age group [25].

## Conclusions

Our findings confirmed the association of cMetS with existence of MetS in the pediatric age group. In addition, its validity was confirmed. This score is becoming widely used in pediatric epidemiological research. Further studies are needed in different populations for using this score in clinical practice.

## Abbreviations

cMetS: continuous metabolic syndrome score
MetS: metabolic syndrome
WC: waist circumference
MAP: mean arterial blood pressure
ROC: receiver operation characteristic
AUC: area under the curve
TG: triglycerides
BP: blood pressure
HDL-C: high density lipoprotein-cholesterol
CASPIAN: Childhood and Adolescence Surveillance and Prevention of Adult Non-communicable Disease
BMI: body mass index
FBG: fasting blood glucose
TC: total cholesterol
LDL-C: low density lipoprotein-cholesterol
